# A Stitch in Time Defeats the Landry-Guillain-Barré Strohl Syndrome

**DOI:** 10.7759/cureus.29047

**Published:** 2022-09-11

**Authors:** Prateek Upadhyay, Richa Saroa

**Affiliations:** 1 Anaesthesia and Intensive Care, Government Medical College & Hospital, Chandigarh, IND

**Keywords:** conduction block in neural tissue, areflexic motor paralysis, intravenous immunoglobulin therapy, fulminant polyradiculopathy, guillain-barre syndrome, intensive care units, polyradiculopathy

## Abstract

The Landry-Guillain-Barré Strohl Syndrome (LGBS) or Guillain-Barré syndrome (GBS) is an acute, frequently severe, potentially fatal, and fulminant polyradiculopathy. It is an autoimmune illness, which usually occurs as a sequela of certain known infections. In this case report, we are discussing the case of a 12-year-old girl who was managed in the ICU for LGBS successfully and recovered promptly. This case highlights the importance of timely administration of intravenous immunoglobulin (IVIG) therapy, which resulted in prompt recovery, reduced duration of ICU stay, and morbidity.

## Introduction

The Landry-Guillain-Barré Strohl Syndrome (LGBS) or Guillain-Barré syndrome (GBS) is an acute, frequently severe, potentially fatal, and fulminant polyradiculopathy. With the eradication of polio, it has turned out to be a common cause of acute neuromuscular paralysis with no specific age group. However, it is more common in the adult population as compared to the pediatric [1]. In this report, we are discussing the case of a 12-year-old girl who was managed in the ICU for LGBS successfully and recovered promptly.

## Case presentation

A 12-year-old girl presented with the chief complaints of fever and cough for seven days, and an inability to stand, speak, or swallow for two days. Fever was documented to be 38.4 degrees Celsius at its maximum. It was not associated with chills, rigors, or rash. There was no diurnal variation and it was relieved temporarily by medication (acetaminophen). The cough was productive, more at night, associated with a sore throat. After five days of illness, the child developed an acute onset of weakness in the lower limbs manifesting as an inability to hold slippers, stand, or walk. The weakness was symmetrical and associated with generalized body aches. There was a gradual weakness in the upper limbs and the ability to phonate to the point of complete loss of speech on the following day. It was followed by an inability to swallow both solids as well as liquids within 48 hours.

The patient denied numbness, tingling sensation, headache, vomiting, altered sensorium, abnormal body movements, ophthalmic involvement, deviation of face, profuse sweating, dizziness, palpitation, inability to pass urine, pain in the abdomen, jaundice, abdominal distention, diarrhea, spinal tenderness, and recent vaccination. There was no significant past medical or surgical history, perinatal history, developmental history, and family history. The child’s immunization status was complete.

On presentation, intravenous fluid resuscitation was performed with lactated ringer, normal saline, and 5% dextrose-normal saline and started on broad-spectrum antibiotics (amoxycillin/clavulanic acid orally 500 mg eight-hourly). The patient was intubated (endotracheal tube internal diameter 6.5 mm, cuffed) in view of airway compromise secondary to excessive oral secretions (bulbar involvement), and maintained on T-piece. Vitals were stable and examination showed a normal fundus. Nerve conduction velocity was also within normal range. CSF culture demonstrated acellular fluid with mildly raised proteins. Additionally, bearing in mind the high possibility of LGBS, a dose of 0.5 grams per kg body weight (25 mg) intravenous immunoglobulin (IVIG) therapy was started on day one itself.

The next day, in view of persistently raised blood pressure more than the 95th centile ( >130/90 mmHg), labetalol infusion was started (0.25 mg/kg/hour). IVIG was continued. (35 mg on day two), The patient was then shifted to ICU for mechanical ventilation in view of progressively deteriorating respiratory efforts. The patient was managed as per ICU protocol and rigorous physiotherapy (range of motion exercises and incentive spirometry) was ensured. The next day, another 30 mg of IVIG was administered over four hours. Shallow respiratory spontaneous efforts started to appear. Finally, on the fifth day, 30 mg IVIG was administered to add up to a total of 120 mg. There was a progressive improvement observed in the clinical examination findings as shown in Table 1.

**Table 1:** Clinical examination showed progressive improvement over six days DTR: deep tendon reflex; Power: motor power

Clinical Examination	Day 1	Day 2	Day 3	Day 4	Day 5	Day 6
Power: Upper Limbs (Shoulder/Elbow/Wrist)	4/5	4/5	4/5	4/5	5/5	5/5
Power: Feet (Ankle)	2/5	1/5	2/5	2/5	3/5	4/5
Power: Legs (Knee)	2/5	2/5	3/5	3/5	4/5	5/5
Thighs (Hip)	3/5	3/5	3/5	4/5	4/5	5/5
DTR	0	0	2	2	3	3
Plantar	Mute	Down	Down	Down	Down	Down

Over the following three days, the patient was gradually liberated from ventilator support followed by tracheal extubation after 48 hours. After gradually tapering off oxygen therapy, she could be shifted to the ward after two days. On follow-up, her MRI of the spine was done and it had normal findings. She was discharged subsequently.

## Discussion

LGBS reveals itself as a rapidly developing areflexic motor paralysis, which can be associated with sensory disturbances. The typical course begins with an ascending paralysis, observed as “rubbery legs”. The basis of clinical manifestation is the conduction block in the neural tissue. It is apparent from the current literature that the illness is a consequence of the molecular mimicry mechanism in which there is an immune response misdirected to nerve tissues [2]. The inciting event is usually a gastrointestinal or respiratory infection and it takes about one to three weeks after the infection for LGBS to show up. The common organisms indicted are *Campylobacter jejuni* and human herpes virus: cytomegalovirus or Epstein Barr virus. Other viruses (HIV, hepatitis E) and *Mycoplasma pneumonia *are also known triggers. Recent immunization has a role to play [2,3].

Although the diagnosis can be made clinically, variant syndromes do necessitate other investigations like nerve conduction velocity and electromyography [4]. It is be noted that serial electrophysiological studies were supportive in establishing the concluding precise diagnosis, but in our case, it somehow showed a normal picture. CSF findings are distinctive with an elevated protein level without pleocytosis and normal or mildly raised cell count (albuminocytological dissociation).

Treatment of GBS is broadly in two simultaneous considerations. Firstly, there’s a need for constant evaluation of the patient in view of anticipated or ongoing intensive care and secondly, there should be prompt initiation of immunomodulating therapy to limit the damage being done by the illness likely by restriction of further nerve injury [5]. Now that the auto-immune aspect of the illness is well proven, based on high clinical suspicion, immune-modulating therapy can be commenced. Currently, the two standard modalities of this are IVIG and plasmapheresis or plasma exchange (PE). IVIG treatment is a tried and tested treatment that expressively hastens recovery from the illness. Likewise, there is a significant improvement in plasma exchange without any serious adverse events [6]. Treatment must be administered within two weeks of illness to have vigorous clinical improvement. However, even if delayed, significant improvement can be seen if started within four weeks [7]. The commonly encountered side effects include headache, flu-like symptoms, myalgia, nausea, etc. In the matter of comparison, IVIG treatment, when promptly commenced within two weeks from the onset has a similar effect as plasma exchange and, showed to be equally efficacious [8]. Upsetting adverse events were not notable with either of the two. IVIG with its ease of administration and lesser patient discomfort is much more likely to be completed than plasma exchange. However, it is to be well known that giving IVIG after having given plasma exchange does not have any added advantage [9].

The plasma fibrinogen level can be taken as a marker that reflects the condition and magnitude of illness. Plasma fibrinogen levels should be measured at least once before and once by the time of the final planned plasma exchange procedure. If plasma fibrinogen comes up to be lowered by 30% or more compared to its level at presentation, it indicates a successful plasma exchange. Whereas, no substantial decrease (<30%) in fibrinogen levels despite plasma exchange necessitates the need to continue the therapy to improve the outcome or diminish the risk of relapse [10]. Coming to other modalities, corticosteroids have been studied for their effect on GBS and it is concluded that when given alone, they neither hasten recovery nor affect the long-term prognosis. Instead, it is noticeable that oral corticosteroids can potentially delay recovery. The adverse effects of corticosteroids (particularly, diabetes requiring insulin) also state against the use of them in GBS [11]. Eculizumab is gaining popularity for its potential use in LGBS. It has shown promising results during clinical trials [12].

While immunomodulation is important for clinical improvement, it’s vital to look for signs and symptoms that warrant ICU care in the illness. There needs to be strict vigilance if the illness shows a fulminant course (onset to admission <7 days), bulbar weakness, and neck flexion weakness. In the presence of these clinical features, ICU care should be initiated/continued and step-down is not recommended [13]. Additionally, when it is being managed in ICU, the patients entail constant monitoring of vitals, adequate nutrition, deep vein thrombosis (DVT) prophylaxis as per ICU protocol, and early consideration of tracheostomy (after two weeks of intubation) and physiotherapy. Frequent turning and attentive bed sore prophylaxis are important. Reassurance is also a must for a good recovery [2]. Pain (neuropathic) has been reported in 33-70% of cases. Gabapentin, carbamazepine, and tricyclic antidepressants can serve as multimodal components to treat pain. Opioid analgesics can be used, but autonomic instability should be considered before administration [14].

Patients usually recover well in the acute form of LGBS and have a favorable long-term prognosis. More than 80% are able to walk without support after six months [15]. Likewise, most (more than 90%) of pediatric patients have favorable recovery irrespective of the severity of illness at admission and electrophysiological subtypes [3]. Dysautonomia and speech impairment, however, blares clarion with tragic outcomes and is an independent risk factor for mechanical ventilation in pediatric patients while those with acute gastrointestinal infection and acute motor axonal neuropathy take longer to recover [16-18]. Mortality during the illness in its acute form is about 5% (3.5-12%) despite advanced critical care [19,20].

## Conclusions

This case highlights the importance of timely administration of IVIG, which resulted in prompt recovery, reduced duration of ICU stay, and morbidity. It also stimulates comparatively and continuous evaluation of the ongoing practice and commencing/modifying the treatment before it's too late. Indeed, a stitch in time saved nine by the timely administration of IVIG.
